# Effect of Fiber Length on Carbon Nanotube-Induced Fibrogenesis

**DOI:** 10.3390/ijms15057444

**Published:** 2014-04-29

**Authors:** Amruta Manke, Sudjit Luanpitpong, Chenbo Dong, Liying Wang, Xiaoqing He, Lori Battelli, Raymond Derk, Todd A. Stueckle, Dale W. Porter, Tina Sager, Honglei Gou, Cerasela Zoica Dinu, Nianqiang Wu, Robert R. Mercer, Yon Rojanasakul

**Affiliations:** 1 Department of Pharmaceutical Sciences, West Virginia University, 1, Medical Center Drive, Morgantown, WV 26506, USA; E-Mails: amanke1@mix.wvu.edu (A.M.); sluanpitpong@hsc.wvu.edu (S.L.); xiaoqinghe6@gmail.com (X.H.); 2 Department of Chemical Engineering, Statler College of Engineering and Mineral Resources, West Virginia University, 395 Evansdale Drive, PO Box 6102, Morgantown, WV 26506, USA; E-Mails: cdong3@mix.wvu.edu (C.D.); cerasela-zoica.dinu@mail.wvu.edu (C.Z.D.); 3 Pathology and Physiology Research Branch, National Institute for Occupational Safety and Health, 1095 Willowdale Rd., Morgantown, WV 26505, USA; E-Mails: lmw6@cdc.gov (L.W.); lob0@cdc.gov (L.B.); rhd8@cdc.gov (R.D.); jux5@cdc.gov (T.A.S.); dhp7@cdc.gov (D.W.P.); sst2@cdc.gov (T.S.); rpm7@cdc.gov (R.R.M.); 4 Department of Mechanical and Aerospace Engineering, Statler College of Engineering and Mineral Resources, West Virginia University, 395 Evansdale Drive, PO Box 6102, Morgantown, WV 26506, USA; E-Mails: honglei.gou@mail.wvu.edu (H.G.); nick.wu@mail.wvu.edu (N.W.)

**Keywords:** carbon nanotubes, fiber length, lung fibrosis, ROS, type I collagen, TGF-β

## Abstract

Given their extremely small size and light weight, carbon nanotubes (CNTs) can be readily inhaled by human lungs resulting in increased rates of pulmonary disorders, particularly fibrosis. Although the fibrogenic potential of CNTs is well established, there is a lack of consensus regarding the contribution of physicochemical attributes of CNTs on the underlying fibrotic outcome. We designed an experimentally validated *in vitro* fibroblast culture model aimed at investigating the effect of fiber length on single-walled CNT (SWCNT)-induced pulmonary fibrosis. The fibrogenic response to short and long SWCNTs was assessed via oxidative stress generation, collagen expression and transforming growth factor-beta (TGF-β) production as potential fibrosis biomarkers. Long SWCNTs were significantly more potent than short SWCNTs in terms of reactive oxygen species (ROS) response, collagen production and TGF-β release. Furthermore, our finding on the length-dependent *in vitro* fibrogenic response was validated by the *in vivo* lung fibrosis outcome, thus supporting the predictive value of the *in vitro* model. Our results also demonstrated the key role of ROS in SWCNT-induced collagen expression and TGF-β activation, indicating the potential mechanisms of length-dependent SWCNT-induced fibrosis. Together, our study provides new evidence for the role of fiber length in SWCNT-induced lung fibrosis and offers a rapid cell-based assay for fibrogenicity testing of nanomaterials with the ability to predict pulmonary fibrogenic response *in vivo*.

## 1. Introduction

Carbon nanotubes (CNTs) have generated great interest commercially with their unique physicochemical properties such as high tensile strength and conductivity [1,2]. However, despite their numerous applications, inhalation of these nanoparticles exerts negative effects on the normal physiological functions of lungs and causes pulmonary toxicity. They are particularly scrutinized given their high aspect ratio similar to asbestos fibers which induce inflammatory and fibrotic lung reactions, pleural mesothelioma and lung cancer [3–5]. Moreover, CNT structure facilitates their entry, deposition and residence in the lungs, resulting in impaired clearance from the lungs [6]. Collectively, these features reinforce the safety concerns about their pathogenicity and potential adverse effects on the health of exposed workers and the general population.

At present, human data regarding fibrogenicity assessment of CNTs is lacking and information on *in vivo* toxicity is limited, especially for single-walled CNTs (SWCNTs). Initial findings demonstrate that SWCNTs are capable of causing rapid and progressive interstitial fibrosis in murine models given their ability to translocate into the surrounding areas in the lung causing inflammation, granulomatous lesions and sub-pleural fibrosis [7–10].

Several factors including morphology, size, shape, surface charge and agglomeration state have been shown to influence the reactivity of SWCNTs [2]. In general, a number of studies have illustrated the CNT length-dependent adverse effects on pleural inflammation and granuloma formation [11,12], cytotoxicity [13], and inflammasome activation [14]. Additionally, fiber length has been shown to dictate multi-walled CNT (MWCNT) retention and clearance from the lungs [12,15]. While recent studies have suggested incomplete phagocytosis as a paradigm for CNT length-mediated toxic effects, the direct effect of SWCNT length on fibrosis and the underlying mechanisms remain to be elucidated.

Recent findings have also demonstrated that SWCNTs can directly interact with interstitial lung fibroblasts to exert their direct fibrogenic effects both *in vivo* and *in vitro* in the absence of persistent inflammation and cell damage [16–18]. The cellular fibrogenic effect of CNTs provides a platform to develop an *in vitro* fibroblast model for assessing the fibrogenic potential of CNTs with various physicochemical properties. The primary objective of our study was to develop a predictive *in vitro* model for assessing the contribution of SWCNT fiber length on fibrogenicity using reactive oxygen species (ROS) generation, collagen expression and transforming growth factor-beta (TGF-β) release as the *in vitro* endpoints of fibrogenic response. Such mechanism-based cell model fibrogenic biomarkers enable *in vitro* risk assessment which can be further validated by the fibrogenic response in animal models. We hypothesize that fiber length is a significant determinant of SWCNT-induced lung fibrosis and that our *in vitro* cultured fibroblast model would be predictive of the fibrogenic response *in vivo*. This model would enable rapid fibrogenicity testing of CNTs and could be used for early detection of oxidative stress response, collagen production and TGF-β release which are potential biomarkers for CNT-induced fibrosis. In this study, we evaluated and compared the *in vitro* cellular toxicity, ROS generation, collagen production and TGF-β release in human lung fibroblasts treated with SWCNTs of different lengths. To validate our *in vitro* model, we performed *in vivo* experiments evaluating the effect of SWCNT length on lung fibrosis in mice.

## 2. Results and Discussion

### 2.1. Physicochemical Characterization of Single-Walled Carbon Nanotubes (SWCNT)

SWCNT samples were characterized using atomic force microscopy (AFM) and energy dispersive X-ray spectroscopy (EDX-S) for size measurements and elemental analysis, respectively. Table 1 provides information on the purity, length and diameter characteristics for the SWCNT samples used in this study. Short and long SWCNTs differed slightly in their diameter but very substantially in their length both in the solution and dry forms. For each SWCNT type, particle lengths were comparable in the solution and dry forms, suggesting that they were efficiently dispersed in the culture medium. Table 2 provides quantitative elemental analysis for the SWCNT samples. Short SWCNTs were 92.82 wt % elemental carbon with 5.7 wt % oxygen, whereas long SWCNTs were 90.9 wt % carbon with 8 wt % oxygen. Both short and long SWCNTs were similar in their elemental composition.

### 2.2. Dose- and Length-Dependent Effects of SWCNTs on Cell Viability and Collagen Expression

**Figure 1.**
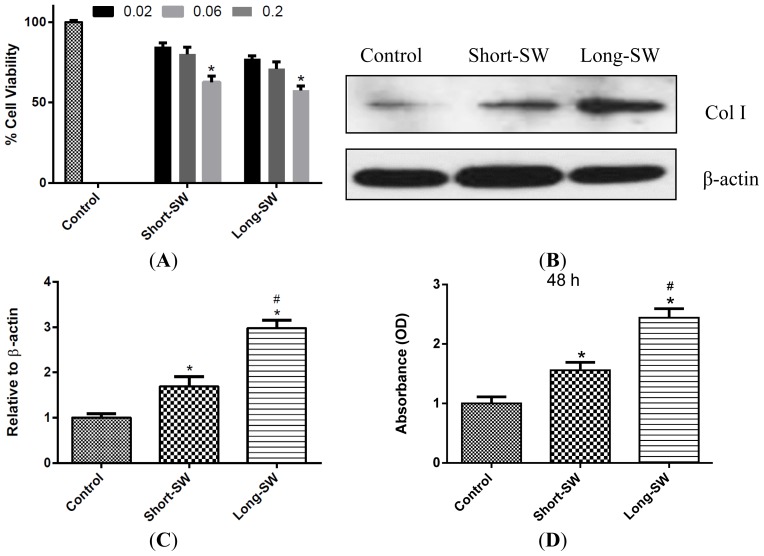
Effect of Single-Walled Carbon Nanotubes (SWCNTs) on cell viability and type I collagen expression. (**A**) Subconfluent cultures of normal human lung fibroblasts (NHLF) cells were exposed to SWCNTs of various lengths for 48 h within the concentration range of 0.02–0.2 μg/cm^2^ and compared to untreated control by WST-1 colorimetric assay; (**B**) Western blots showing length-dependent effect of SWCNTs on collagen I production. Subconfluent cultures of NHLFs were treated with SWCNTs with various lengths for 48 h and analyzed for collagen I expression by Western blotting. Blots were reprobed with β-actin antibody to confirm equal loading of the samples. The immunoblot signals were quantified by Image J.; (**C**) Relative protein quantification via Image J.; (**D**) NHLFs were treated with SWCNTs for 48 h at 0.06 μg/cm^2^ and analyzed for soluble collagen content by Sircol^®^ assay. Values are mean ± S.D. (*n* = 3); * *p* < 0.05 as compared to untreated control; # *p* < 0.01 *vs.* Short-SW only.

Cultured normal human lung fibroblasts (NHLF; Lonza, Walkersville, MD, USA) were exposed to short and long SWCNTs and analyzed for cellular toxicity. This study was performed to optimize the experimental doses of SWCNT that are relevant to *in vivo* lung fibrosis. Lung fibroblasts were treated with different concentrations of SWCNT for 48 h and analyzed for cell viability by WST-1 assay. The doses of 0.02–0.2 μg/cm^2^ were used in this study since they are physiologically relevant and derived from pulmonary exposure data in mice, *i.e.*, 10–80 μg/mouse which corresponds to 0.02–0.16 μg/cm^2^ of mouse lung alveolar surface area [7,16,18]. Both short and long SWCNTs induced a dose-dependent decrease in cell viability of the cultured fibroblasts (Figure 1A). At equal dosing, long SWCNTs induced slightly more cellular toxicity than short SWCNTs, although the difference was not statistically significant under the test conditions. We also investigated the fibrogenic or collagen-inducing effect of SWCNTs in lung fibroblasts. To avoid the interfering effect of cell toxicity on collagenic activity of the cells, we performed experiments using low-dose (0.06 μg/cm^2^) SWCNTs. Figure 1B,C shows that at this dose both long and short SWCNTs induced a substantial increase in collagen expression as determined by Western blot assay. Analysis of soluble collagen content by Sircol^®^ assay confirmed the result and indicated the collagenic activity of SWCNTs (Figure 1D). Long SWCNTs were substantially more fibrogenic than short SWCNTs based on the Western blot and Sircol^®^ results.

### 2.3. SWCNTs Induced Cellular Oxidative Stress and Fibrogenic Response

**Figure 2.**
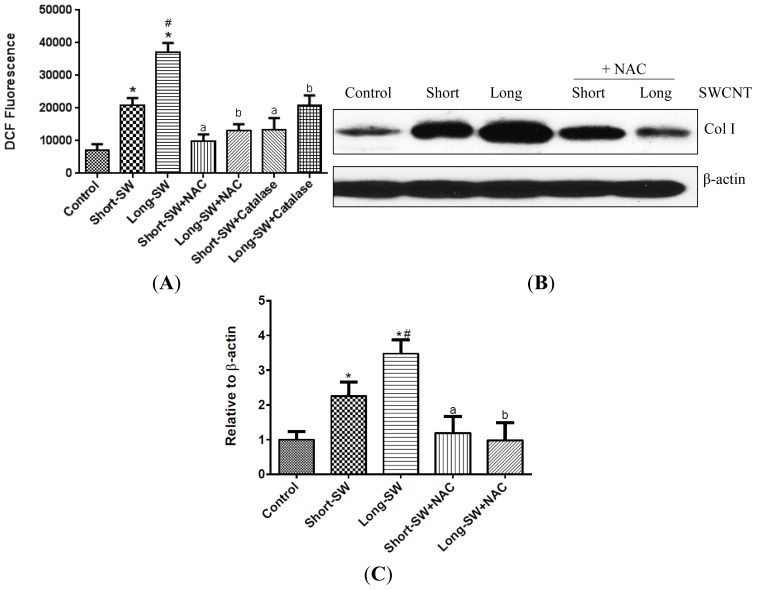
Effect of SWCNT length on reactive oxygen species (ROS) response. (**A**) After treatment with different SWCNTs at 0.06 μg/cm^2^, cells were incubated with dichlorodihydrofluorescein (DCF) dye and fluorescence intensity as a measure of oxidative stress was read at 2 h post-treatment. Prior to treatment, NHLF cells were also pretreated for 1 h with NAC (*N*-acetyl cysteine; 10 mM) or catalase (1000 U/mL) and then analyzed for ROS production by measuring DCF fluorescence; (**B**) Subconfluent cultures of NHLF were pretreated with NAC for 1 h and treated with SWCNT of different lengths at 0.06 μg/cm^2^ and analyzed for type I collagen by Western blotting; (**C**) NHLFs were pretreated with NAC for 1 h and later exposed to SWCNTs for 48 h at 0.06 μg/cm^2^. The resulting cell lysates were analyzed for soluble collagen content by Sircol^®^ assay. Plots are mean ± S.D. (*n* = 4); * *p* < 0.05 as compared to untreated control; # *p* < 0.01 *vs.* Short-SW only; a, *p* < 0.05 compared to Short-SW only; b, *p* < 0.05 compared to Long-SW only.

Since oxidative stress has been implicated as an underlying mechanism for pulmonary fibrosis [9], we investigated the effect of SWCNT length on cellular ROS generation as an indication of oxidative stress. Cells were treated with long and short SWCNTs and analyzed for ROS generation by fluorometry using dichlorodihydrofluorescein diacetate (DCF-DA) as a fluorescent probe. Both short and long SWCNTs were found to dramatically increase the cellular DCF fluorescence intensity as compared to control level (Figure 2A). Long SWCNTs were more potent than short ones in inducing the ROS generation (Figure 2A). Pretreatment of the cells with antioxidant *N*-acetyl cysteine (NAC) or with peroxide scavenger catalase strongly inhibited the ROS-inducing effect of both long and short SWCNTs (Figure 2A,B). The antioxidant pretreatment also inhibited the collagen-inducing effect of both long and short SWCNTs, supporting the role of ROS in the fibrogenic process (Figure 2B,C).

### 2.4. Effect of SWCNT Length on TGF-β Expression and Secretion

**Figure 3.**
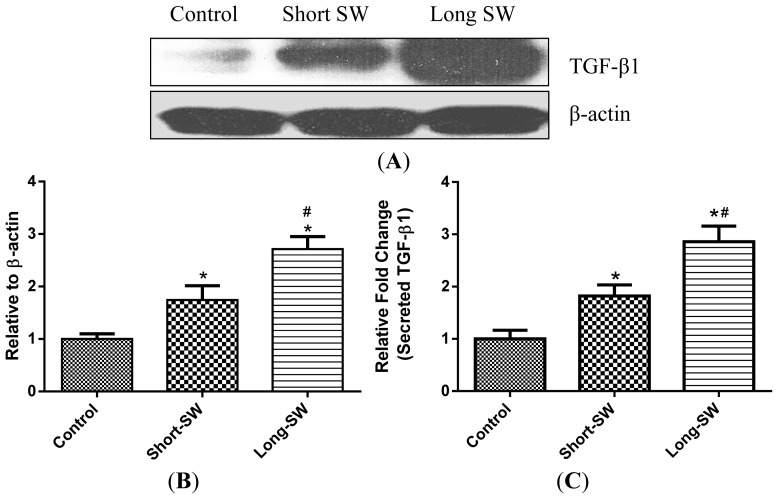
Effect of SWCNT fiber length on TGF-β expression. NHLF cells were exposed to 0.06 μg/cm^2^ of SWCNTs for 48 h. (**A**) Endogenous TGF-β levels in cell lysates were measured by Western blotting; (**B**) Relative TGF-β levels were quantified by Image J.; (**C**) Secreted TGF-β levels in the treated cell supernatants were measured by enzyme-linked immunosorbent assay (ELISA). Values are mean ± S.D. (*n* = 3); * *p* < 0.05 *vs.* non-treated control; # *p* < 0.05 *vs.* Short-SW only.

TGF-β is one of the key regulators of lung fibrosis and increased expression of TGF-β has been consistently reported in biopsies of fibrotic lungs [19]. To investigate the effect of SWCNT length on TGF-β production, lung fibroblasts were exposed to short and long SWCNTs, and analyzed for TGF-β expression by Western blotting. Consistent with the trend observed with the ROS and collagen effects, both long and short SWCNTs were able to induce TGF-β expression over control level with the effect being more pronounced with the long SWCNTs (Figure 3A,B). To confirm this finding, we analyzed TGF-β level in the supernatants of treated and control cells by enzyme-linked immunosorbent assay (ELISA). In good agreement with the Western blot results, the ELISA results indicated an increased secretion of TGF-β by the cells in response to long SWCNT treatment as compared to short SWCNT or control treatment (Figure 3C).

### 2.5. Role of Reactive Oxygen Species (ROS) in SWCNT-Induced TGF-β Expression

**Figure 4.**
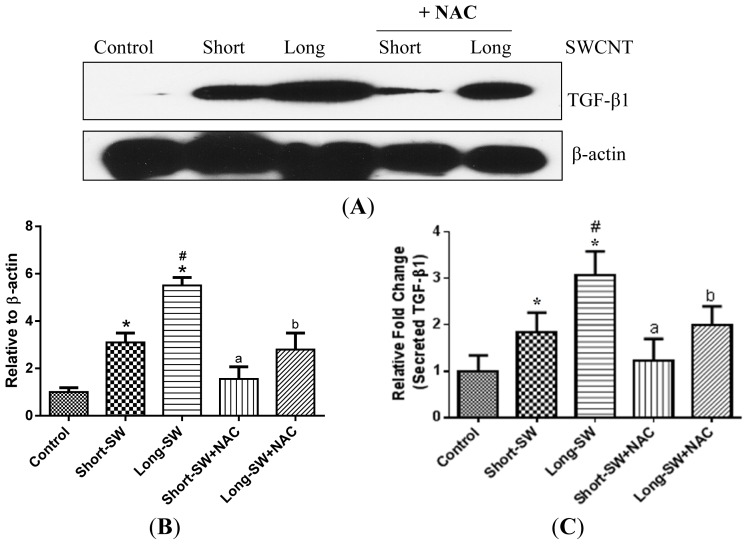
Effect of ROS on SWCNT-induced TGF-β expression. (**A**) Subconfluent cultures of NHLF were pretreated with NAC for 1 h and treated with SWCNT of different lengths at 0.06 μg/cm^2^ and analyzed for TGF-β by Western blotting; (**B**) Protein quantification using Image J.; (**C**) Effect of NAC on secreted TGF-β levels in the treated cell supernatants measured by ELISA. Values are mean ± S.D. (*n* = 3); * *p* < 0.05 *vs.* nontreated control; # *p* < 0.05 *vs.* Short-SW only; a, *p* < 0.05 compared to Short-SW only; b, *p* < 0.05 compared to Long-SW only.

ROS has been shown to drive TGF-β-mediated cellular responses [20]; however, its role in SWCNT-induced TGF-β expression has not been demonstrated. To determine whether ROS is involved in the upregulation of TGF-β by SWCNTs, lung fibroblasts were exposed to long and short SWCNTs in the presence or absence of antioxidant NAC, and their effect on TGF-β expression was determined by Western blotting. Figure 4A,B shows that NAC was able to inhibit the TGF-β upregulation by SWCNTs, both long and short forms. Analysis of secreted TGF-β in the treated cell supernatants by ELISA similarly indicated the inhibitory effect of NAC on SWCNT-induced TGF-β release (Figure 4C), thus confirming the role of ROS in SWCNT-induced TGF-β upregulation.

### 2.6. In Vivo Validation of the Pulmonary Fibrogenic Effect of SWCNTs

**Figure 5.**
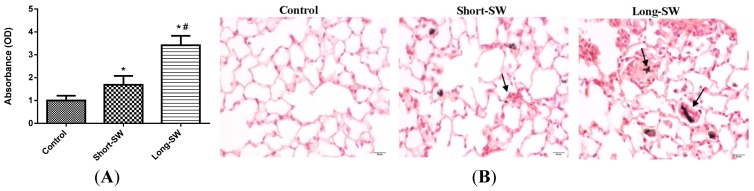
Effect of SWCNT length on fibrogenic response *in vivo*. Mice were exposed to 50 μL of dispersed SWCNT (40 μg/mouse) via pharyngeal aspiration for 90 days after which the animals were sacrificed and the lungs were isolated, lysed and analyzed for (**A**) soluble collagen content by Sircol^®^ assay; (**B**) histopathology after Sirius red staining. Scale bar = 20 μm; Arrows denote the thickening of collagen fibers around the CNT; Values are means ± S.D., (*n* = 5 mice per group); * *p* < 0.05 *vs.* BSA/dipalmitoyl phosphatidylcholine (DPPC) treated control; # *p* < 0.05 *vs.* Short-SW only.

To validate the *in vitro* length-dependent fibrogenic effect of SWCNTs, mice were exposed to short and long SWCNTs via pharyngeal aspiration and analyzed for lung fibrosis by Sircol^®^ collagen assay and histopathology. An occupationally relevant dose of 40 μg/mouse, and exposure time of 3 months were used to ensure a robust fibrogenic response based on previous *in vivo* findings [3,21,22]. Lung collagen content as determined by Sircol^®^ assay was substantially upregulated in the SWCNT-treated mice as compared to control mice (Figure 5A). Long SWCNTs induced a higher fibrogenic response than short SWCNTs, consistent with the *in vitro* finding (Figure 1B–D). Figure 5B shows representative light micrographs of Sirius Red stained lung sections from control and three-month short and long SWCNT aspiration. The histopathological study, evaluated by Sirius red staining, confirmed the biochemical findings indicating greater accumulation and thickness of collagen fibers only in the SWCNT exposed mouse lung sections. As compared to the alveolar section observed from BSA treated control, both short and long SWCNT fibers were found to induce collagen fibers which were condensed around SWCNT deposited areas (observed throughout the alveolar interstitial space).

Long SWCNT treatment demonstrated a highly fibrotic lung response compared to short SWCNT as evidenced by the dense lesions containing abundant collagen (denoted by arrows). Thus, histopathological analysis confirmed the results and indicated greater fibrogenicity of the long CNTs over short CNTs (Figure 5B). In good agreement with our *in vitro* results, both long and short SWCNTs upregulated TGF-β expression over control level with the effect being more pronounced with the long SWCNTs *in vivo*. Together, these results validated our *in vitro* model and indicated fiber length as a key determinant of SWCNT fibrogenicity.

### 2.7. Discussion

Lung fibrosis induced by SWCNTs has been well documented [16,22,23] but the effect of specific SWCNT properties on lung fibrosis remains controversial and largely undefined [9,24]. Revealing the physicochemical properties influencing CNT fibrogenicity is essential due to the promise surrounding CNT exploitation. Although a few studies have reported the length effect of MWCNT on lung toxicity, the effect of SWCNT length on lung fibrosis has not been reported. Additionally, the mechanism underlying CNT-induced fibrosis remains to be elucidated. Several potential mechanisms of CNT-induced fibrosis have been suggested, including pre-existing inflammation [8], epithelial mesenchymal transition [25], pro-fibrogenic mediators [26], and oxidative stress [27]. Our previous research data showed the fibrogenic effect of CNTs based on their direct interaction with collagen-producing fibroblasts [16,17]. The present study was designed to evaluate the effect of fiber length on SWCNT-induced fibrosis and to develop an *in vitro* model to predict the fibrogenic response *in vivo*.

We reported here that SWCNTs can induce collagen expression in both *in vitro* and *in vivo* models (Figures 1 and 5). Long SWCNTs were more potent inducer of collagen expression than short CNTs as determined by Western blot and Sircol^®^ assays. The collagen-inducing effect of SWCNTs was not due to their proliferative activity since fibroblast cell growth was not increased by the SWCNT treatment as indicated by WST-1 assay (Figure 1A). Moreover, all collagen expression data presented in this study were normalized against β-actin or cellular content as described in the Experimental Section. Since collagen deposition is a hallmark of lung fibrosis, cellular collagen content could be used as a functional assay for nanoparticle fibrogenicity *in vitro*. The consistency of the *in vitro* and *in vivo* results observed in this study supports the validity of the *in vitro* model for prediction of CNT fibrogenicity.

CNTs have been shown to induce ROS generation in various cell types [28–31]. ROS-dependent activation of transcription factors and signaling pathways has also been shown to regulate fibrosis [32]. It is generally accepted that the presence of transition metal impurities such as iron and nickel contributes to the oxidative stress and fibrotic responses to CNTs [29,30,33]. In this study, we used well characterized CNTs with known metal impurities to study the effects of fiber length on ROS generation and fibrosis. The elemental analysis indicated low iron content for both short (0.12 wt %) and long (0.13 wt %) SWCNTs compared to those reported in the previous studies. Besides, no significant difference was observed in the elemental composition of the two SWCNT samples used in this study (Table 2). Compared to control, both short and long SWCNTs induced a stronger oxidative stress response (Figure 2). Long SWCNTs elicited a more robust ROS response than short SWCNTs, indicating a length-dependent effect not associating with metal content. Variances within short and long SWCNT-induced ROS response could be attributed to their differential cellular uptake as the larger load of internalized CNTs may be responsible for increased disruption of the integrity of cell membrane and intracellular organelles [34]. However, CNT uptake may not be the only factor in the enhanced toxicity of long SWCNTs. The failure of cells to entirely engulf the long fibers results in prolonged phagocytic oxidative outburst. We believe that the “frustrated” phagocytosis might be one of the mechanisms involved in the differential ROS response exerted by SWCNTs used in our study [6,11,12]. Thus, long SWCNTs may be more likely to accumulate within the cells compared to the shorter length CNT, causing a greater build up within the cell thereby disrupting the cell membrane and cell organelles and eliciting increased toxicity in the form of robust ROS generation. Moreover, the difference observed between the short and long SWCNT could be attributable to different cellular signaling pathways targeted by the two CNTs [35]. The higher toxicity of our long SWCNTs could also be explained based on lipid peroxidation following the interaction between cell membrane and long SWCNT [36]. However, these phenomena responsible for the pronounced length-dependent ROS response need to be further elucidated. Our study also showed that ROS played a significant role in SWCNT-induced fibrogenesis as evidenced by the inhibition of collagen and TGF-β production by the antioxidant NAC (Figures 2 and 4). Thus, ROS generation may be used as a rapid screening test for CNT fibrogenicity assessment.

An interesting result from this study was the ROS-dependent activation of TGF-β by SWCNTs. Both long and short forms of SWCNT upregulated the expression of TGF-β (Figure 3), the effect that is dependent on ROS generation (Figure 4). ROS has been shown to play a role in TGF-β-mediated fibroblast to myofibroblast differentiation with the differentiated cells serving as an additional source of ROS generation [37]. TGF-β is one of the most potent fibrogenic mediators known to stimulate collagen production by fibroblasts [38]. SWCNTs elicited length-dependent TGF-β activation as assessed by Western blotting (Figure 3A,B) and ELISA (Figure 3C), suggesting its potential utility as a biomarker for CNT-induced fibrosis.

There were discrepancies observed in the collagen I and TGF-β responses along with pre-treatment with NAC. While our study findings demonstrated the importance of ROS in CNT-induced collagen production, it is not the only determining factor and there may be alternate mechanisms or additional factors that contribute to the observed effect. For instance, the fibrogenic effect observed with short and long SWCNTs could be due to activation of entirely two distinct cellular pathways. To illustrate this, a study showed that CNT exposure stimulated a length dependent activation of TGF-β/Smad2/collagen III signal transduction [39]. In addition, the variance between collagen I production among short-SW + NAC and long-SW + NAC in our study did not reach statistical significance in Figure 2B,C. Similarly, CNT-induced TGF-β is dependent on ROS, but other factors such as matrix metalloproteinases contribute towards TGF-β activation [40]. Likewise, SWCNT-induced inflammatory cascade has been shown to elevate TGF-β levels *in vivo* [7].

An additional key finding from this study was the association of the *in vitro* and *in vivo* results, indicating the potential usefulness of the *in vitro* model as a predictive screening tool for testing of fibrogenicity testing of nanomaterials. The three-month aspiration study in mice demonstrated a length-dependent pathogenicity *in vivo* as evidenced from the histopathological and biochemical findings. However, the complexity of *in vivo* environment also requires factoring in the CNT retention within the pleural space and clearance from the lung since these materials are bio-persistent and resist degradation. MWCNTs have been shown to undergo length-dependent retention within the pleural space followed by a subsequent fibrotic response [12]. Exposure to intact (individual length 5.9 μm) and ground MWCNT (0.7 μm) revealed a size-dependent deposition with agglomerates of intact MWCNT within the upper airways, while ground MWCNTs dispersed throughout the lung tissue [41]. A more recent finding demonstrated the biopersistence of MWCNTs within the lung up to 336 days of exposure with 95% of the initial lung burden still persistent in the alveolar region [42]. Though, our current study did not report quantitative tests for clearance and persistence of SWCNTs with different lengths and further work needs to be done to address the role of fiber length on SWCNT clearance *in vivo*.

Fibroblasts are the main cellular source of collagen production in the lung whose accumulation characterizes lung fibrosis. Currently, there is an urgent need for efficient *in vitro* models for fibrogenicity testing of nanomaterials. The large and rapidly expanding number of engineered nanomaterials makes it impossible to test them all in animals due to time constraints and prohibitive cost. In this study we developed and tested a fibroblast cell assay as a low cost, predictive *in vitro* model that allows rapid assessment of multiple endpoints critical to the development of lung fibrosis.

## 3. Experimental Section

### 3.1. SWCNT Preparation

SWCNTs were prepared by plasma purified chemical vapor deposition process and were obtained from Cheap Tubes Inc. (Brattleboro, VT, USA). They were dispersed in culture medium containing 5% serum by water-bath sonication. Before exposure to the cells, the SWCNT dispersion was lightly sonicated (Sonic Vibra Cell Sonicator, Sonic & Material Inc., Newtown, CT, USA) with the power, frequency, and amplitude settings of 130 W, 20 kHz, and 60% respectively for 10 s.

### 3.2. Chemicals and Reagents

Antibodies for collagen type I and TGF-β were obtained from Fitzgerald (Concord, MA, USA) and Cell Signaling Technology, Inc. (Beverly, MA, USA), respectively. β-Actin antibody and horseradish peroxidase (HRP)-conjugated secondary antibodies were obtained from Santa Cruz Biotechnology (Santa Cruz, CA, USA). The antioxidant catalase was obtained from Roche Molecular Biochemicals (Indianapolis, IN, USA). The oxidative probe 2′,7′-dichlorodihydrofluorescein diacetate (H_2_DCF-DA) and the antioxidant *N*-acetyl cysteine (NAC) were obtained from Sigma Chemical Inc. (St. Louis, MO, USA).

### 3.3. Energy Dispersive X-ray Spectroscopy (EDX-S)

EDX-S was used to perform elemental analysis of SWCNT samples. Data were collected on a LEO 1530 VP scanning electron microscope equipped with an energy-dispersive X-ray analyzer (Hitachi S-4700 Field Emission Scanning Electron Microscope, Hitachi High Technologies Co., Tokyo, Japan). A few drops of SWCNT dispersion in cell culture medium were placed on a silicon wafer and allowed to air-dry. The silicon wafer was then mounted on an aluminum stub for EDX-S analysis.

### 3.4. Atomic Force Microscopy (AFM)

AFM was used to measure the length and diameter distribution of SWCNT samples using Digital Instrument Nanoscope II (Model No. MFP-3D-AFM, Asylum Research, Goleta, CA, USA). A Si tip (50–90 kHz AC240TS, Asylum Research, Goleta, CA, USA) was used to perform tapping mode in air. SWCNT samples were deposited on mica surfaces (9.5 mm diameter, 0.15–0.21 mm thickness, Electron Microscopy Sciences, city, state, USA) and allowed to dry overnight under vacuum. Scan angel was set as 0, scan rate was set as 0.5 Hz, and resolution was set as 512. Scan images of 20 × 20 or 10 × 10 μm areas were acquired. For each sample, at least 30 individual SWCNTs were counted and measured to obtain average length and diameter distribution.

### 3.5. Cell Culture

Normal human lung fibroblasts (NHLFs) were obtained from Lonza (Walkersville, MD, USA). The cells were maintained in Fibroblast Basal Medium (Lonza, CC-4126, Walkersville, MD, USA) containing growth supplements. The cells were cultured at 37 °C in 5% CO_2_ incubator and were passaged at preconfluent densities using a medium containing 0.05% trypsin.

### 3.6. Cytotoxicity Assay

Cytotoxicity assay was carried out using WST-1 cell viability assay kit (Roche Molecular Biochemicals, Indianapolis, IN, USA) as per the manufacturer’s instructions. Cells were plated in triplicate in 96-well plates at the density of 2.0 × 10^4^ cells/well in CS-C medium. Following overnight culture, the cells were incubated with the indicated concentrations of SWCNT for 24 and 72 h. After incubation, WST-1 reagent was added and the cells were incubated for an additional 4 h. The plates were then read at the wavelength of 420 nm using a microplate reader (Model 3550; BioRad, Richmond, CA, USA).

### 3.7. Sircol^®^ Collagen Assay

Soluble collagen content was determined by Sircol assay^®^ (Biocolor Ltd., Belfast, UK), according to the manufacturer’s protocol. Briefly, lung fibroblasts (1 × 10^5^ cells/well) were cultured in 6-well plates and treated with SWCNTs of different lengths at the indicated concentrations for 24 and 48 h. Equal amount of Sirius red reagent (Biocolor Ltd., Belfast, UK) and cell culture supernatant (50 μL) were added together and mixed for 30 min. The collagen-dye complex was then precipitated by centrifugation at 13,000× *g* for 5 min, washed with ethanol, and dissolved in 0.5 M NaOH. A 200 μL aliquot of the mixture was transferred to a 96-well plate and analyzed for optical absorbance at 540 nm.

### 3.8. Western Blot Analysis

Collagen protein expression was determined by Western blotting. After specific treatments, cells were incubated in lysis buffer containing 20 mM Tris–HCl (pH 7.5), 1% Triton X-100, 150 mM sodium chloride, 10% glycerol, 1 mM sodium orthovanadate, 50 mM sodium fluoride, 100 mM phenylmethylsulfonyl fluoride, and a commercial protease inhibitor mixture (Roche Molecular Biochemicals, Indianapolis, IN, USA) at 4 °C for 20 min. Cell lysates were collected and protein concentrations were determined using a bicinchoninic acid protein assay kit (Pierce Biotechnology, Rockford, IL, USA). Equal amount of protein per sample (40 μg) was resolved under denaturing conditions by 10% SDS-PAGE and transferred onto a nitrocellulose membrane. The membranes were blocked for 1 h in 5% nonfat dry milk in TBST (25 mM Tris–HCl, pH 7.4, 125 mM sodium chloride, 0.05% Tween 20) and incubated with appropriate primary antibodies at 4 °C for 12 h. Membranes were washed thrice with TBST for 10 min and incubated with HRP-labeled isotype-specific secondary antibodies for 1 h at room temperature. The immune complexes were then detected by enhanced chemiluminescence detection system (Supersignal^®^ West Pico, Pierce, Rockford, IL, USA). The bands were quantified via densitometry using Image J. software, version 10.2 (GraphPad Software Inc., La Jolla, CA, USA). Mean densitometry data from independent experiments were normalized to results in cells from control experiments.

### 3.9. DCF Fluorometric Assay for ROS Detection

Cellular ROS production was determined fluorometrically using H_2_DCF-DA as a fluorescent probe. After treatment with SWCNTs, cells were incubated with the probe (5 mM) for 30 min at 37 °C, after which they were analyzed for fluorescence intensity using a multi-well plate reader (FLUOstar OPTIMA BMG LABTECH Inc., Durham, NC, USA) at the excitation/emission wavelengths of 485/590 nm.

### 3.10. TGF-β Enzyme-Linked Immunosorbent Assay (ELISA)

Cells were plated in 6-well plates at the density of 2 × 10^5^ cells/well in culture medium and incubated overnight before the cells were subjected to treatment. After the treatment, cell supernatants were collected and analyzed for TGF-β level using a commercial ELISA kit (#KAC1688, Invitrogen, Camarillo, CA, USA) as per manufacturer’s protocol. Briefly, cell samples or reference standards (100 μL) were added to the wells of a microplate that was pre-coated with TGF-β monoclonal antibody and incubated for 2 h at room temperature. After washing unbounded substances, a HRP-conjugated polyclonal antibody against TGF-β was added to the wells and incubated for 2 h at room temperature. After washing and addition of 100 μL of substrate solution, optical density was determined on a microplate reader (FLUOstar OPTIMA BMG LABTECH Inc., Durham, NC, USA) at 450 nm.

### 3.11. SWCNT Animal Model

Pathogen-free male C57BL/6J mice (Jackson Laboratories, Bar Harbor, ME, USA) weighing 25–30 g were used in this study. Animals were housed in an “Association for Assessment and Accreditation of Laboratory Animal Care” (AAALAC)-accredited, specific-pathogen-free, environmentally controlled facility at National Institute for Occupational Safety and Health (NIOSH). All experimental procedures were conducted in accordance with a protocol (#11-LR-M-018) approved on 26 July 2011 by the Institutional Animal Care and Use Committee (IACUC). The animals were treated with SWCNTs by pharyngeal aspiration. Briefly, animals were anesthetized by an intraperitoneal injection of ketamine and xylazine (45 and 8 mg/kg) and placed on a board in the supine position. The animal’s tongue was extended with padded forceps. A suspension of the test material (40 μg/50 μL per mouse) was placed on the back of the tongue. A slight pull of the tongue results in a reflex gasp and aspiration of the droplet. The tongue was held, and the animal was monitored for a few breaths after aspiration. At 90 days post-exposure, mice were sacrificed and lung tissues were isolated, homogenized, lysed and analyzed for collagen content by Sircol^®^ assay. For histopathology studies, paraffin-embedded lung sections were stained with Sirius red and examined under a light microscope.

### 3.12. Statistical Analysis

The data represent mean ± S.D. from three or more independent experiments. ANOVA was performed to determine statistical significance between treatment and control groups using Graph Pad Prism 6.0 (GraphPad Software Inc., La Jolla, CA, USA) at a confidence level of * *p* < 0.05.

## 4. Conclusions

**Figure 6.**
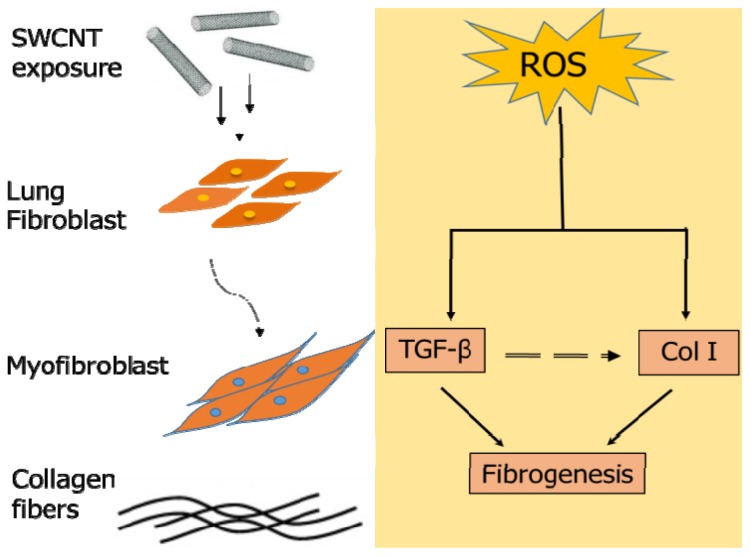
Schematic representation of mechanism involved during SWCNT-induced fibrogenesis. Reactive oxygen species (ROS) play a key role in SWCNT-induced collagen (Col I) and transforming growth factor-beta (TGF-β) expression (solid arrows). ROS mediate collagen I and TGF-β upregulation, thereby inducing fibrogenesis (solid arrows). Furthermore, upregulation of TGF-β in turn increases collagen production via fibroblast and myofibroblast proliferation (dashed arrow).

The present study demonstrated the length-dependent effect of SWCNTs on ROS generation, TGF-β expression and collagen content in cultured human lung fibroblasts. Long SWCNTs induced substantially more ROS, TGF-β and collagen production than short SWCNTs independent of the effect of metal impurities. Figure 6 is a schematic representation of the mechanism involved in response to SWCNT exposure and the interdependent relationship between collagen I, ROS and TGF-β and their interplay in fibrogenesis. ROS played a key role in SWCNT-induced collagen and TGF-β expression. The *in vivo* finding confirmed the robust fibrogenic response induced by long SWCNTs *in vitro*, supporting the predictive value of the *in vitro* model and suggesting fiber length as an important determinant of SWCNT fibrogenicity. The *in vitro* model could serve as a rapid high-throughput screen for fibrogenicity testing of other nanomaterials and offers a low cost alternative to animal models.

## Figures and Tables

**Table 1.** Physicochemical Characterization of SWCNTs. The table describes the purity, diameter and length distribution measured via AFM.

SWCNT type	Purity	Length (μm)		Diameter (nm)

		Solution form	Dry form	
Long	>90%	12.31 ± 0.53	13.4 ± 0.62	11.3 ± 6.20
Short	>90%	1.13 ± 0.39	0.89 ± 0.21	10.8 ± 5.41

**Table 2.** Physicochemical Characterization of SWCNTs. Elemental distribution measured via EDX-S.

Element	SWCNT type	

	Short (wt %)	Long (wt %)
C	92.82	90.9
O	5.77	8
Al	0.06	0.01
Si	0.06	0.08
S	0.11	0.1
Cl	0.3	0.2
Ca	0.1	0.12
Cr	0.16	0.31
Fe	0.13	0.12
Co	0.48	0.1
Mg	–	0.04
